# 4G mobile phone radiation alters some immunogenic and vascular gene expressions, and gross and microscopic and biochemical parameters in the chick embryo model

**DOI:** 10.1002/vms3.1273

**Published:** 2023-09-19

**Authors:** Md. Sadequl Islam, Md. Mominul Islam, Md. Moshiur Rahman, Khaleda Islam

**Affiliations:** ^1^ Department of Anatomy and Histology Faculty of Veterinary and Animal Science Hajee Mohammad Danesh Science and Technology University Dinajpur Bangladesh; ^2^ Department of Pathology and Parasitology Faculty of Veterinary and Animal Science Hajee Mohammad Danesh Science and Technology University Dinajpur Bangladesh; ^3^ Graduate School of Biomedical and Health Sciences Hiroshima University Hiroshima Japan; ^4^ Institute of Nutrition and Food Science University of Dhaka Dhaka Bangladesh

**Keywords:** 4G mobile radiation, biochemical, chick embryo model, immunogenic, vascular gene, histopathological

## Abstract

**Background:**

The risks to human health have grown over the past 10 years due to the excessive use of mobile phones.

**Objectives:**

The study was designed to determine the harmful effects of 4G mobile phone radiation on the expression of immunogenic and vascular genes and gross, microscopic and biochemical alterations in the development of chicken embryos.

**Methods:**

Sixty individuals in the exposure group were subjected to mobile phones with a specific absorption rate of 1.4 W/kg and a frequency of 2100 MHz positioned at a distance of 12 cm in the incubator for 60 min/night for 14 days. The histopathological examination involved hematoxylin and eosin staining, whereas cresyl violet staining was used to evaluate the condition and number of neurons in the brain. The biochemical parameters of amniotic fluid were analysed using the photometry method, and the expression of *VEGF‐A* and immunity genes (*AvBD9, IL6*) was measured using the real‐time PCR (qPCR) technique.

**Results:**

Compared to the control, the exposure group's body weight and length significantly decreased (*p* < 0.05). Subcutaneous bleeding was seen in the exposure group. Urea, creatinine, alkaline phosphatase, aspartate aminotransferase and alanine aminotransferase levels were all significantly higher than in the control group (*p* < 0.05). The exposed group showed pathological lesions in the liver and degenerated neurons with lightly stained nuclei in the cerebral cortex. Hyperchromatic neurons were significantly higher in the exposure group (58.8 ± 2.28) compared to the control (6.6 ± 0.44) (*p* < 0.05). 4G exposure reduced lymphocyte count in the caecal tonsil (86.8 ± 5.38) compared to the control (147.2 ± 9.06) (*p* < 0.05). Vascular gene *mRNA* expression was higher, but immune gene expression was lower in the exposed group.

**Conclusion:**

Exposure to mobile phone radiation may result in gross, microscopic and biochemical changes, as well as alterations in gene expression that could hinder embryonic development.

## Graphical abstract

Exposure to 4G significantly reduced body weight and length, elevated liver enzyme levels, pathological liver lesions, hyperchromatic neurons, decreased lymphocyte count and altered vascular and immune gene expression compared to the control group.

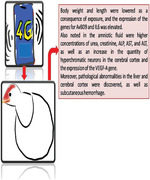

## 1 INTRODUCTION

The rapidly increasing use of 4G mobile phones has raised concerns about the possible health effects of exposure to the electromagnetic radiation emitted by these devices. Many studies have examined the impact of mobile phone radiation on biological systems in recent years. Still, the specific effects on immunogenic and vascular gene expressions and gross and microscopic parameters remain relatively unexplored. The past decade has seen increased human health hazards linked to uncontrolled smartphone use (Fathi & Farahzadi, 2014). Human bodies absorb radiation, converted into electric and magnetic fields, altering the structure of cells, the plasma membrane and receptors for various biomolecules during embryonic development (Al‐Qudsi & Azzouz, 2012; Bhat, 2013). Radiation damages embryonic stem cells, with children and fetuses being more vulnerable than adults (Kheifets et al., 2005; Leitgeb, 2008; Maisch, 2003; Schuz 2010; Siddiqi et al., 2013). Using cell phones during pregnancy and post‐partum may lead to behavioural issues in newborns, according to research by Divan et al. (2008) and Abramson et al. (2009). Additionally, D'Silva et al. (2017) found that 2G and 3G mobile phone radiations can cause structural defects and DNA damage in the liver of chick embryos.

Extended exposure to high‐frequency radiation produced by 2G or 3G cell phones reduces testosterone synthesis and the amount of Sertoli and Leydig cells and causes microscopic changes to seminiferous tubule epithelium (Mugunthan et al., 2015). Long‐term mobile phone use has been linked to various health issues, including insomnia, depression, migraines and sleep deprivation. Prolonged exposure to radiofrequency radiation can harm blood constituents, biochemical parameters and their function and has been associated with high blood pressure, DNA damage, cancer, miscarriage and hormonal imbalances (Kaur et al., 2016). Radiofrequency exposure generates free radicals that produce biochemical, immunological and cancerous effects on the body. Radiofrequency electromagnetic fields (RF‐EMFs) alter gene expression in living cells, including those involved in stress regulation, the cytoskeleton, the cell cycle and the extracellular matrix of skin fibroblasts (Mailankot et al., 2009). Because people tend to hold their cell phones close to their heads during calls, the brain is more vulnerable to radioactivity than other body organs (Irmak et al., 2002).

The chick embryo model is a suitable biological model for studying the effects of radiation, as it resembles the human embryo in its molecular, cellular and anatomical makeup. It also undergoes rapid development in the early stages, making it easy to observe changes, and has relatively large structures (Vergara & Canto‐Soler, 2012). As such, the chick embryo model enables researchers to quickly complete studies and observe every developmental stage (Al‐Qudsi & Azzouz, 2012).

The widespread excessive use of cell phones has left people unaware of the potential effects of EMFs. Exposure to cell phone radiation (4G) during pregnancy has been linked to developmental defects, delayed growth and modifications to the biochemical properties of amniotic fluid. Harmful agents can alter the process of gene expression. This investigation aimed to examine the impact of 4G mobile radiation (2100 MHz) on the expression of specific vascular and immunity genes. There are no existing reports that provide information on the impact of 4G (2100 MHz) mobile phone radiation on the biochemical changes of amniotic fluid, the development of the brain, the vital mucosa‐associated lymphoid organ, such as the caecal tonsil, as well as on the expression of some vascular and immunity genes of chicken embryos.

## 2 MATERIALS AND METHODS

**FIGURE 1:**
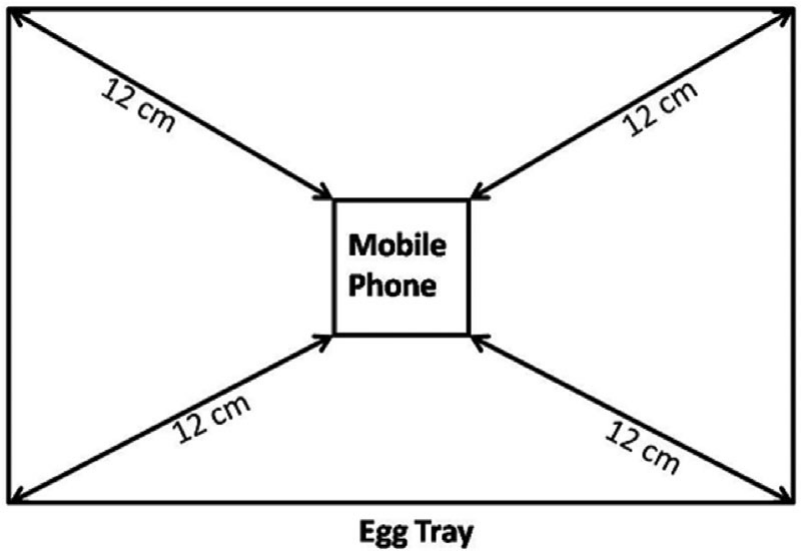
The experimental set‐up involved placing the mobile phones at the centre of the egg tray, with the farthest eggs located 12 cm from the mobile phone.

To experiment, 120 fertile Indian River breed chicken eggs were used. These eggs were collected from VIP Shahadat Hatchery located in Rangpur‐5400 and had an average weight of 60 g. The 60 eggs were separated into two groups. During the testing period, the greatest care was taken to guarantee that the incubator used for the development of the application was separated from any possible EMF impacts that the surrounding environment may have caused. To accomplish this goal, the incubator was put inside a chamber specifically built to provide electromagnetic shielding and was given a regulated atmosphere. Two mobile phones (Samsung Galaxy J5 at specific absorption rate – SAR value 1.4 W/kg) were placed in the middle of the two exposure trays of egg group (A) in the incubator (Figure 1), and a TriField Meter, model 100XE, was used to verify the phones’ EMF output. Two Samsung Galaxy J5 phones with 1.4 W/kg SAR were placed in the incubator's exposure trays for the experiment. The SAR of each chicken embryo in Group A was not calculated. Instead, determining the average exposure level that all of the embryos in Group A experienced using the cell phone's SAR value. Using this strategy, it was possible to keep the exposure environment for both the exposed and control groups under control and consistent. This approach made it possible to compare the two groups meaningfully while minimizing any potential confounding variables and guaranteeing the accuracy of the results. The mobile phones received a video call from a different device for 15 min/h, four times each night (7.00 PM–3.00 AM), with an interval of 1 h between calls while inside the incubator. The silent mode was activated, and the call was scheduled and auto‐received during the 14 days, with an exposure time of 60 min daily.

Conversely, the control group B was not exposed to an EMF, and no mobile device was present in the incubator. The incubator maintained an automatic humidity, temperature and forced air ventilation system. The eggs were placed in unique egg holders that rotated once every 2 h at a fixed rate of 12/day. Regular checks on the growth of embryos were conducted using an egg candler, whereas the humidity and temperature were set at 60% and 37.5°C, respectively. We also thoroughly observed and documented temperature variations in the 4G‐exposed (Group A) and control (Group B) chicken eggs. The incubator's automated temperature control maintained 37.5°C throughout the trial. This temperature was monitored and adjusted to keep the eggs stable throughout 4G mobile phone radiation exposure.

**Group A**: Exposure to 4G (2100 MHz) cell phone radiation. (Application of 4G: High‐speed application‐video conferencing, mobile TV.)

**Group B**: Control group free from any cell phone radiation exposure.

### 2.1 Specimen collection

The Ethical Committee of the Institute of Research and Training (IRT), Hajee Mohammad Danesh Science and Technology University, has approved the strategy (Ref. No. HSTU/IRT/3909) for maintaining chicken embryos in laboratory research and collecting samples. During incubation, samples were taken on the 7th, 10th, and 14th days. A total of 10 samples were collected for each of these days from each group. On the 14th day, a sample of amniotic fluid was taken and centrifuged to evaluate biochemical parameters, including enzyme activity for alkaline phosphatase (ALP), aspartate aminotransferase (AST) and alanine aminotransferase (ALT), along with the levels of urea and creatinine (CT). The embryos collected on days 7, 10 and 14 were euthanized using a painless chilling method after being washed with saline. To examine them, the eggs were cut open at the large end. Total body weights were measured in grams using a digital weight measuring scale (Radwag Wagi Electroniczne, Made in Poland, Model: AS 220.R2, S/N: 544687, 0.1 mg to 220 g), whereas body length was measured in centimetres using a measuring scale. Samples were taken from specific visceral organs: the liver, brain, caecal tonsil and extra‐embryonic membrane. The liver, brain and caecal tonsil samples were preserved in 10% formalin for histopathology.

### 2.2 Histopathological studies

Tissues were dehydrated with increasing alcohol concentrations (70%, 80%, 90%, 95% and 100%) after the formalin had been removed. Each alcohol grade underwent 1 h long dehydration. Following the protocol, the tissues were transferred to xylene‐1 and ‐2 for 90 min each (Islam et al., 2019). After 90 min in liquid paraffin at 60°C, the tissues were set and made into paraffin blocks. The US‐made LEICA RM2125 RTS microtome sliced the paraffin blocks into 6‐μm slices. The paraffin portions were stretched by floating in a 45°C water bath. Next, the stretched tissues were put on clean, grease‐free glass slides and dried in a 62°C hot air oven for 20 min. The dry slides were stained with haematoxylin and eosin (H&E) and covered with a coverslip using adhesive Canada balsam (Drury 1983). An Amscope (MA500) coupled to a Richter Ptica biological microscope, Model: U‐2T, took high‐quality microphotographs of chosen tissues under 10×, 40× and 100× microscopic lenses to better display the findings.

Cresyl violet stained the cerebral cortex to distinguish healthy and sick neurons. Three 5‐min xylene treatments deparaffinized brain slices. After that, the slices were rehydrated for 5 min in phases with decreasing ethyl alcohol concentrations (100%, 95%, 80%, 70% and 50%). After 5 min in deionized water, the slices were stained for 20 min with 0.1% cresyl violet aqueous solution. For optimal staining, the slices were differentiated in 70% alcohol. The slices were dehydrated twice with 90% and 100% ethanol and washed twice with xylene for 5 min to finish staining. After air‐drying, the slices were covered with Canada balsam (Joy et al., 2018).

TCapture was used to count lymphocytes in caecal tonsils and normal and sick neurons in brain slices. A prior work (Madhyastha et al., 2002) utilized a cell field of a certain length to count cells. Each group measured five slides to count cells. Degenerated neurons were hyperchromatic, deformed, pyknotic, shrunken and had an unclear membrane between the cytoplasm and nucleus. Normal neurons have spherical nuclei and well‐defined shapes.

### 2.3 Evaluation of biochemical parameters of amniotic fluid

On day 14, the amniotic fluid was biochemically examined using an 18‐ga syringe (*n* = 5 samples from each group). The air sac and membranes of the eggshell were removed, and the supernatant was centrifuged at 3000*g* for 15 min to remove amniotic fluid. Amniotic fluid samples were tested for urea, CT, ALP, AST and ALTs using photometry (Siemens Advia 1800).

### 2.4 Evaluation of vascular gene *VEGF‐A* and immunity gene expression due to 4G mobile phone exposure

VEGF‐A expression was evaluated using qPCR with GAPDH as the housekeeping gene (Khosravi et al. 2018). At day 14, the chick embryo's extraembryonic membrane was ground and homogenized with liquid nitrogen to obtain RNA. The NANODROP ONE (Thermo Scientific) measured RNA purity and concentration, and the ProtoScript II First Strand cDNA Synthesis Kit (Cat No: E6560S, Biolabs Inc.) synthesized cDNA. The SYBR Green assay Luna Universal qPCR Master Mix (Cat No: M3003S, Biolabs Inc.) and EcoTM 48 Real‐Time PCR (PCR max, ST15 0SA) were used for the qPCR reaction. The thermal profile involved maintaining the temperature at 95°C for 1 min, denaturing for 10 s, annealing for 15 s and extending for 20 s at 72°C. RNA was collected from the chick embryo's caecal tonsils on day 14 for immune gene expression. In the thermal profile employed for qPCR analysis of immune genes, the temperature was held at 95°C for 2 min, denatured for 5 s, annealed for 30 s and extended for 30 s at 72°C. Using ACTB as the housekeeping gene, qPCR evaluated AvBD9 and IL6 expression (Laptev et al. 2019).

### 2.5 Statistical analysis

The statistical approaches that were used in this investigation included the utilization of SPSS version 16 software (SPSS, Inc.), which was used to conduct a normality test, and Student's *t* test in order to analyse the collected data. The data were examined using the normality test so that the assumptions of the *t* test could be validated, and the distribution of the data could be determined. Following that, Student's *t* test was carried out to ascertain whether the differences between the groups were statistically significant. In this particular investigation, a level of statistical significance was determined to exist when *p* was less than 0.05. This threshold suggests a 5% possibility of getting the observed findings by chance alone.

## 3 RESULTS

### 3.1 Congenital disorders

**FIGURE 2:**
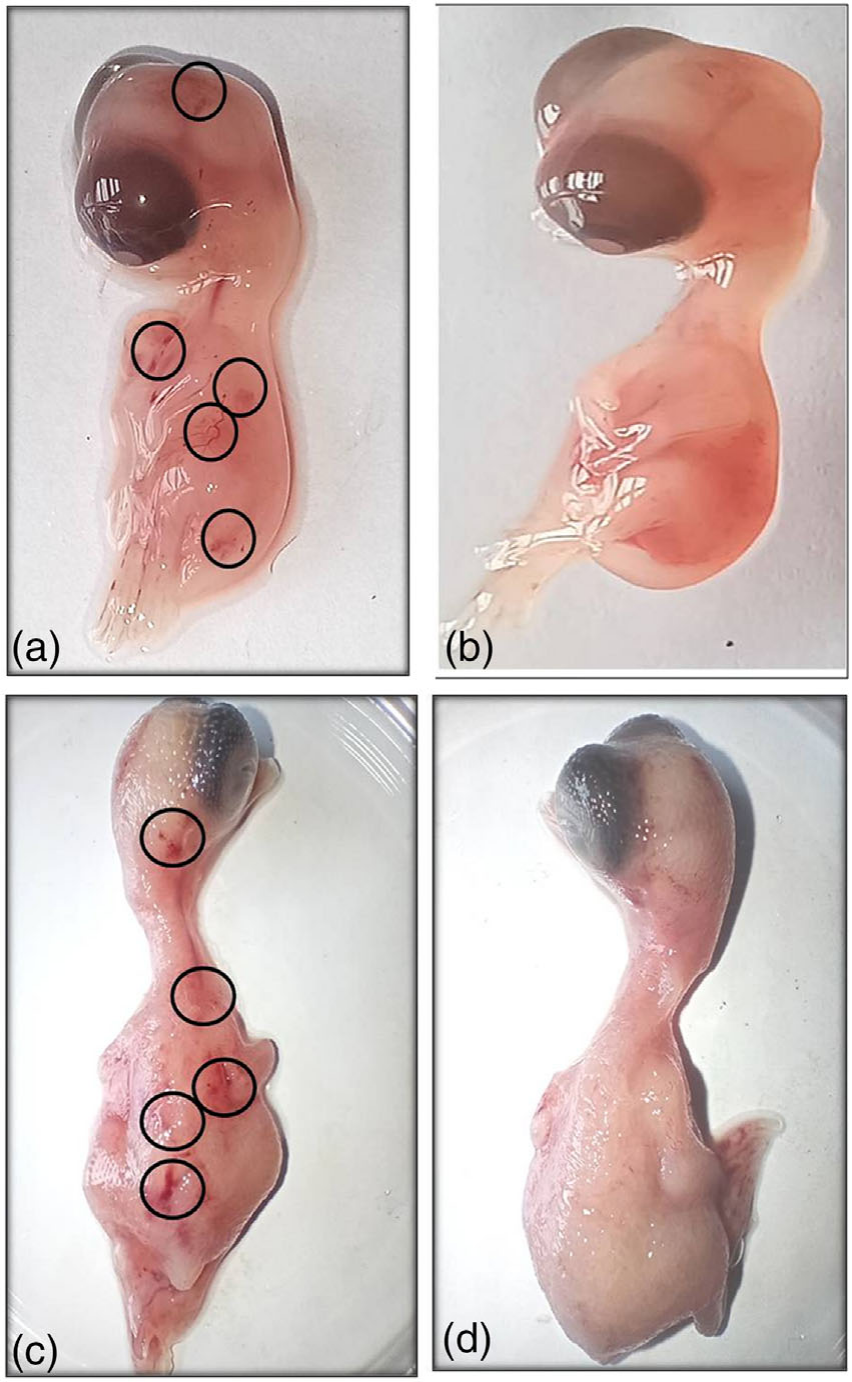
The figure illustrates the 7th and 10th‐day chick embryos of the 4G exposed group (a and c), respectively, displaying subcutaneous haemorrhages marked with circles. In contrast, the control group (b and d) exhibited no signs of haemorrhage.

The exposed embryos (Figure 2a,c) exhibited delayed growth compared to the control group (Figure 2b,d). Figure 2 displays subcutaneous haemorrhages observed in several chick embryos of the 4G exposed groups on days 7 and 10. No other congenital abnormalities or gross malformations were detected in this study.

### 3.2 Growth parameters

**FIGURE 3:**
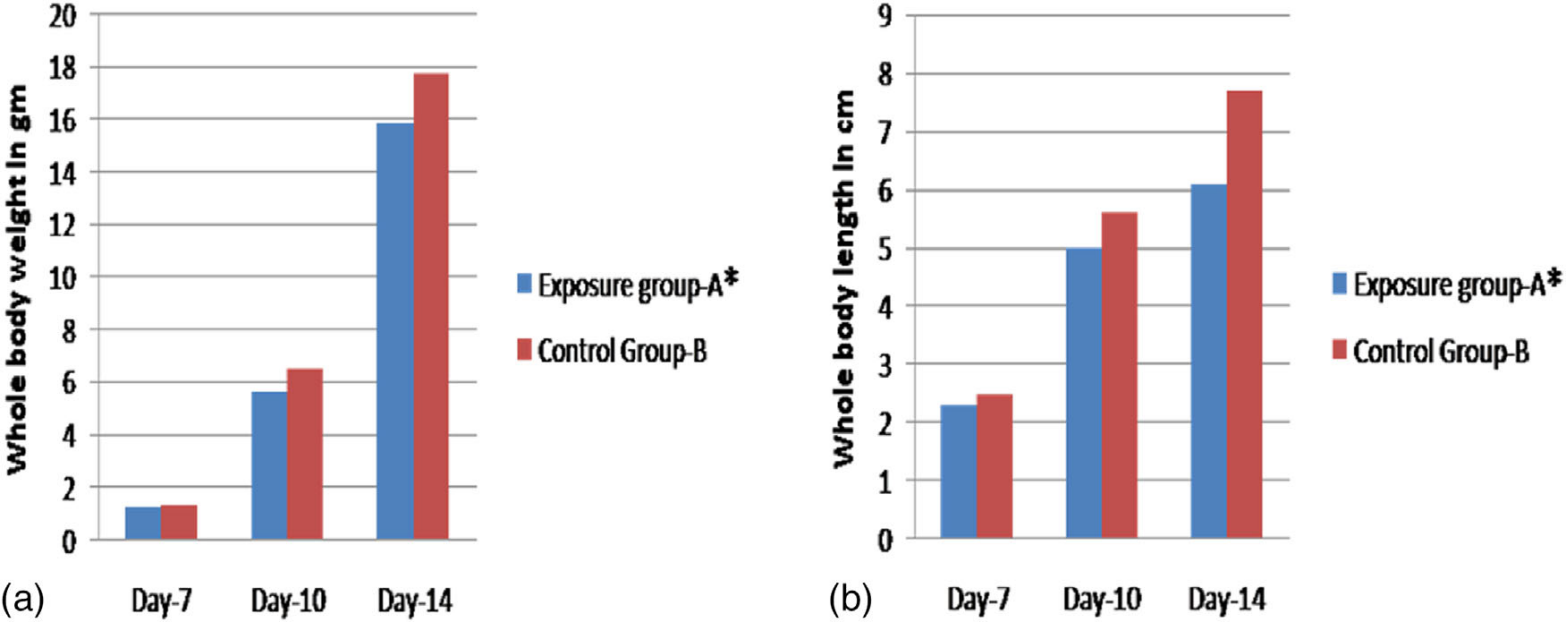
The figure displays the effects of 4G mobile exposure on morphometric measurements of chick embryos, with part (a) representing whole body weight and part (b) representing whole body length. The exposed group (*) exhibited a significant decrease in total body weight and length compared to the control group. The means and standard errors were calculated using *n* = 10 samples for each day group, and both treatment and control were significant at *p* < 0.05.

At days 7, 10 and 14 of exposed embryos, a significant decrease in whole body weight (Figure 3a) and whole body length (Figure 3b) were observed compared to the controls. In group A of days 7, 10 and 14 chick embryos, 4G exposure to mobile radiation resulted in significant retardation of growth in whole body weight compared to controls (Figure 3). The total body weight and length of the exposed group decreased significantly. The means and standard errors were calculated using *n* = 10 samples for each day group. The treatment and control were at *p* < 0.05.

### 3.3 Biochemical parameters of the amniotic fluid

Table 1 demonstrates notable changes in the biochemical parameters of the amniotic fluid of chick embryos following exposure to 4G cell phone radiation. The levels of urea, CT, ALP, AST and ALT significantly increased compared to the control group, with a *p*‐value <0.05.

**TABLE 1:** Biochemical parameters of amniotic fluid in chicken embryos exposed to 4G mobile radiation on day 14 (*n* = 4).

Biochemical parameters	Exposure group A	Control group B	*p*‐Value
Urea (mg/dL)	15.84 ± 1.32	8.26 ± 0.51	0.00002
Creatinine (mg/dL)	0.46 ± 0.014	0.418 ± 0.026	0.0064
ALP (IU/L)	281.99 ± 11.26	5.48 ± 0.40	0.00001
AST (IU/L)	157.442 ± 6.5.13	8.766 ± 0.822	0.00001
ALT (IU/L)	53.65 ± 4.71	3.18 ± 0.82	0.00001

Abbreviations: ALP, alkaline phosphatase; ALT, alanine aminotransferase; AST, aspartate aminotransferase.

### 3.4 Effects on liver development

**FIGURE 4:**
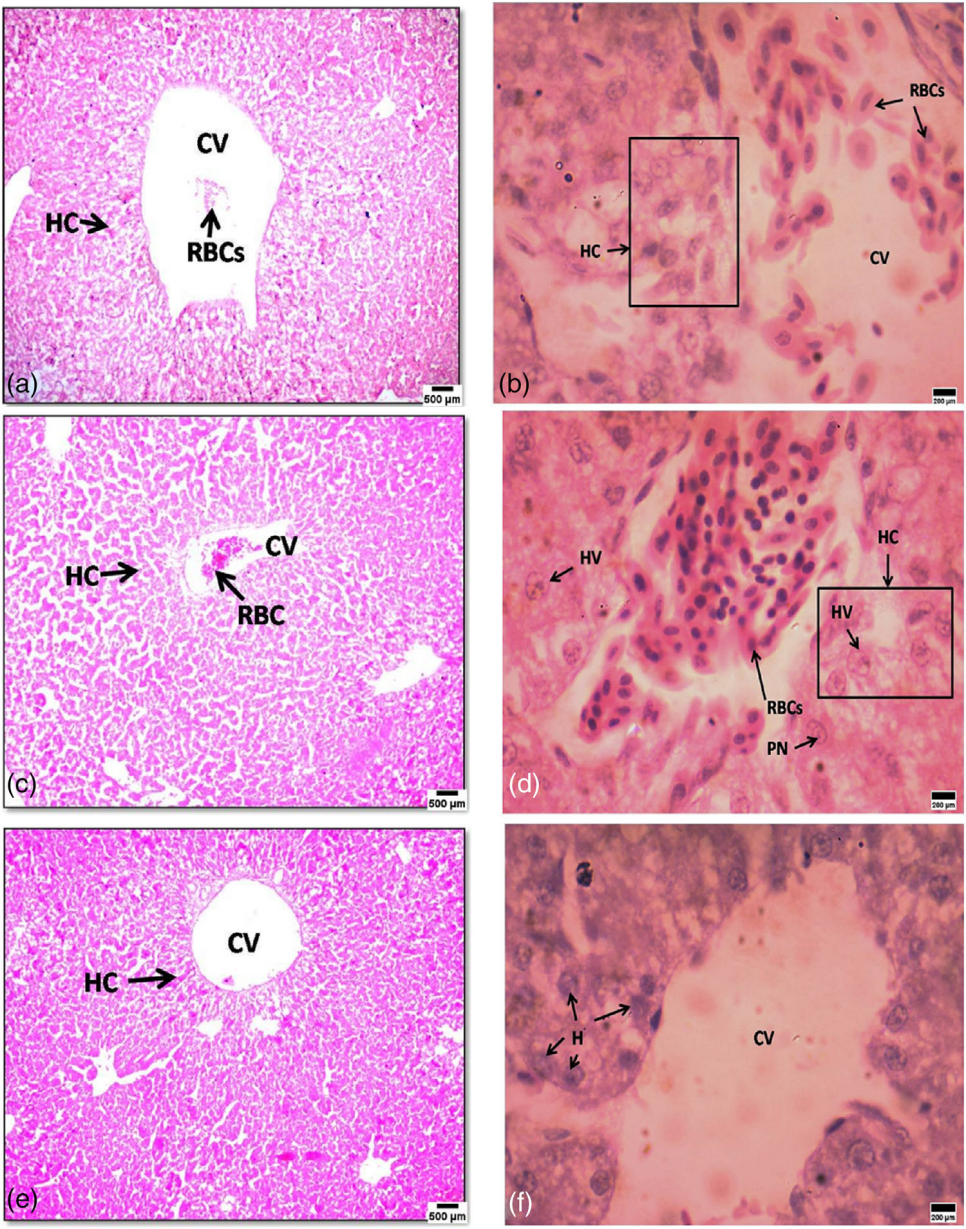
This image shows 14‐day‐old chick embryo liver histopathology. Parts (a) and (c) show the liver of the exposed group to 4G radiation (haematoxylin and eosin [H&E] stained, 10×, scale bar = 500 μm), with the central vein (CV) containing red blood cells and hepatic cords arranged non‐radially around the CV; parts (b) and (d) show the liver of the exposed group, 100×, scale bar = 200 μm, with pyknotic nuclei (PN) and hepatocytes with cytoplasmic vacuolation (HV); part (e) shows the control group without exposure (H&E stained, 10×, scale bar = 500 μm); part (f) shows hepatic cords organised radially around the CV and very evident (H&E stained, 100×, scale bar = 200 μm).

The liver had the right and left lobes in the control chick embryos, with the right being larger. Gross liver cross‐sections in the exposure group exhibited more venous canal haemorrhage on day 14. Exposure also reduced the connective tissue capsule and hepatic cords. Histological investigation of the liver on day 14 in the 4G exposure group (Figure 4a,c) (H&E staining, 10×) showed that central veins (CVs) had red blood cells, and hepatocytes were not distributed radially around the CV. At 100× magnification (Figure 4b,d), the exposed group had pyknotic nuclei (PN) and hepatocytes with cytoplasmic vacuolation (HV). The control group without exposure (H&E stained, 10× and 100×) had hepatic cords organized radially around the CV and no abnormalities (Figure 4e,f).

### 3.5 Effects on brain development

**FIGURE 5:**
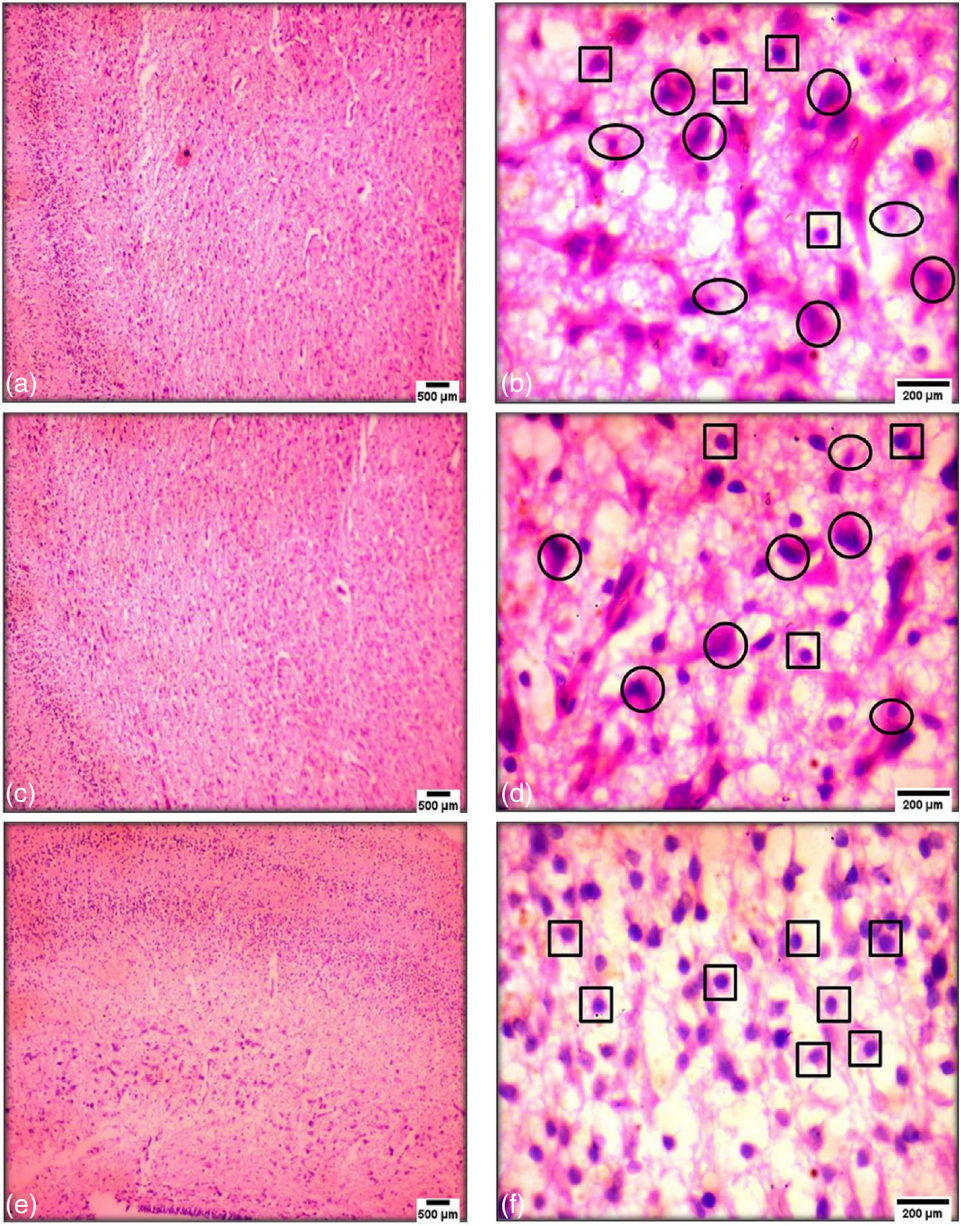
Day 14 histology of the chick embryo's cerebral cortex. Panels (a) and (c) show 4G radiation‐exposed neurons with dark nuclei (hematoxylin and eosin [H&E] stained, 10× magnification, scale bar = 500 μm). Panels (b) and (d) show the 4G radiation‐exposed group (H&E stained, 100× magnification, scale bar = 200 μm), showing degenerated neurons with misshapen, undifferentiated borders between the cytoplasm and the nucleus with darkly stained nuclei (circled), healthy neurons with lightly stained nuclei (circled) and normal astrocytes with a small nucleus (oval). Panel (e) shows weakly stained healthy neurons in the unexposed control group (H&E stained, 10× magnification, scale bar = 500 μm). Panel (f) depicts the control group without exposure (H&E stained, 100× magnification, scale bar = 200 μm) with healthy neurons and weakly stained nuclei (squared).

After staining with H&E, the chick embryo cerebral cortex was studied under a microscope on day 14. 4G radiation‐exposed individuals (Figure 5a–d) showed several conclusions. Degenerated neurons had darkly stained nuclei, malformed and undifferentiated cytoplasm–nucleus boundaries and decreased healthy neurons with faintly stained nuclei. Normal astrocytes had a tiny nucleus. The control group contained mildly stained healthy neurons without exposure (Figure 5e,f).

**FIGURE 6:**
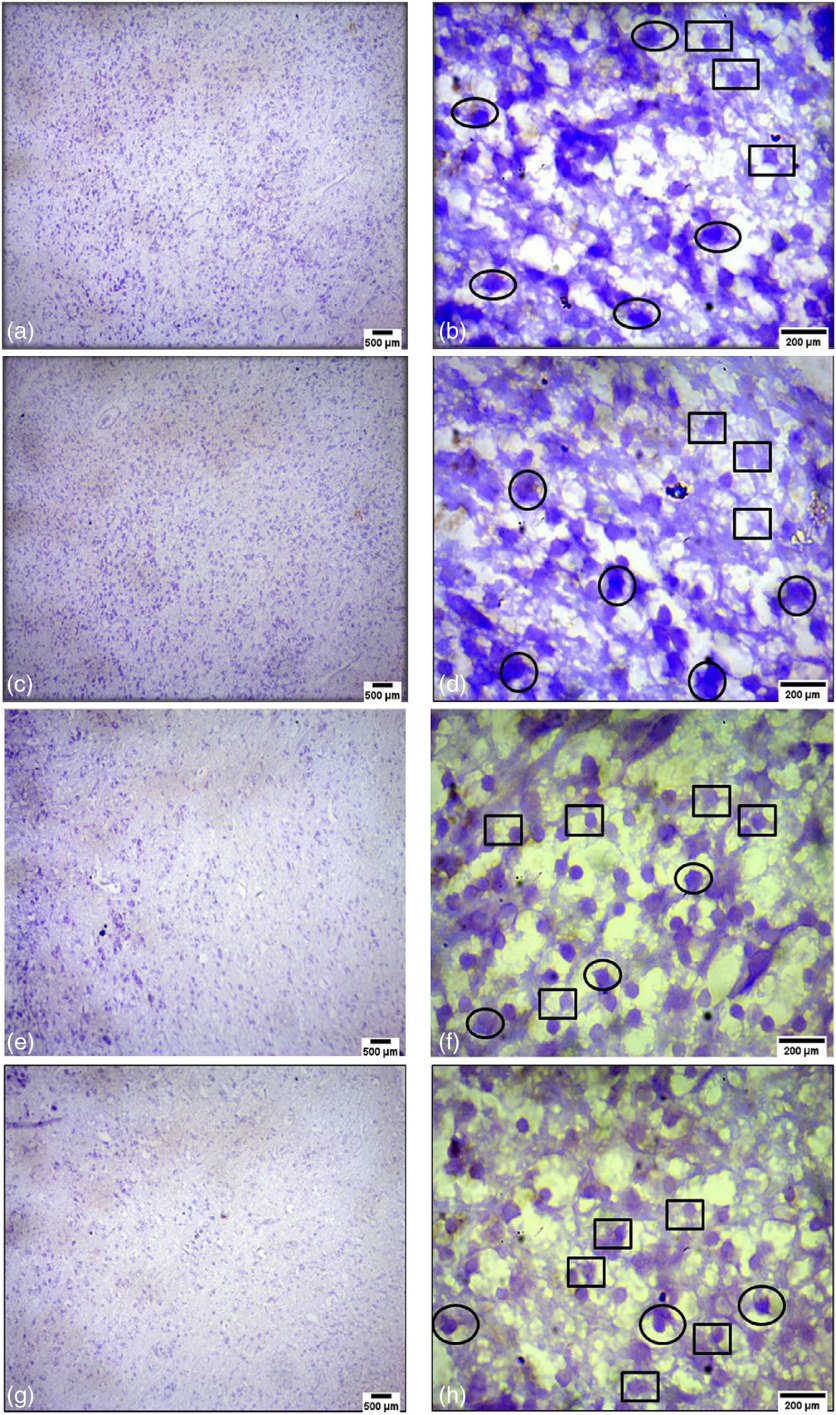
Showing 14‐day chick embryo's cerebral cortex of brain histology. (a and c) Exposed group with 4G radiation (stained with cresyl violet, 10×, scale bar = 500 μm), degenerated neurons with darkly stained nuclei; (b and d) exposed group with 4G radiation (stained with cresyl violet, 100×, scale bar = 200 μm), misshapen, undifferentiated borders in between cytoplasm and the nucleus, shrinked, pyknotic, and hyperchromatic neurons (in oval), normal neurons with reduced in number (in rectangle); (e and g) control group without any exposure (stained with cresyl violet, 10×, scale bar = 500 μm), lightly stained healthy neuron; (f and h) control group without any exposure (stained with cresyl violet, 100×, scale bar = 200 μm), healthy neurons with lightly stained nuclei (in rectangle), unhealthy neuron (in oval).

On day 14, cresyl violet staining was used to count the chick embryo's cerebral cortex's healthy and hyperchromatic neurons. Table 2 shows the number of hyperchromatic and healthy cells in control and 4G‐exposed brains. The exposure group had more hyperchromatic neurons (58.8 ± 2.28) than the control group (6.6 ± 0.44) (*p* < 0.05). The exposure group had fewer healthy neurons (16.4 ± 1.26) than the control group (39.8 ± 5.86) (*p* < 0.05). Cresyl violet stained neuronal cell cytoplasm and nuclei. Radiation‐exposed embryos (Figure 6a–d) had few healthier, more irregular‐shaped neurons than the control group (Figure 6e–h). The exposure group had unevenly organized neurons. Unlike the control group, degenerated neurons with shrunken, PN were deeply stained with cresyl violet.

**TABLE 2:** Comparison of healthy and hyperchromatic neuron counts between exposure (A) and control group (B).

Group	Exposure group (A)	Control (B)
Healthy neurons	16.4 ± 1.26^*^	58.8 ± 2.28^*^
Hyperchromatic neurons	39.8 ± 5.86^*^	6.6 ± 0.44^*^

*Note*: *n* = 5 for each group.

* *p* < 0.05 statistically significantly.

### 3.6 Effects on the development of caecal tonsil

**FIGURE 7:**
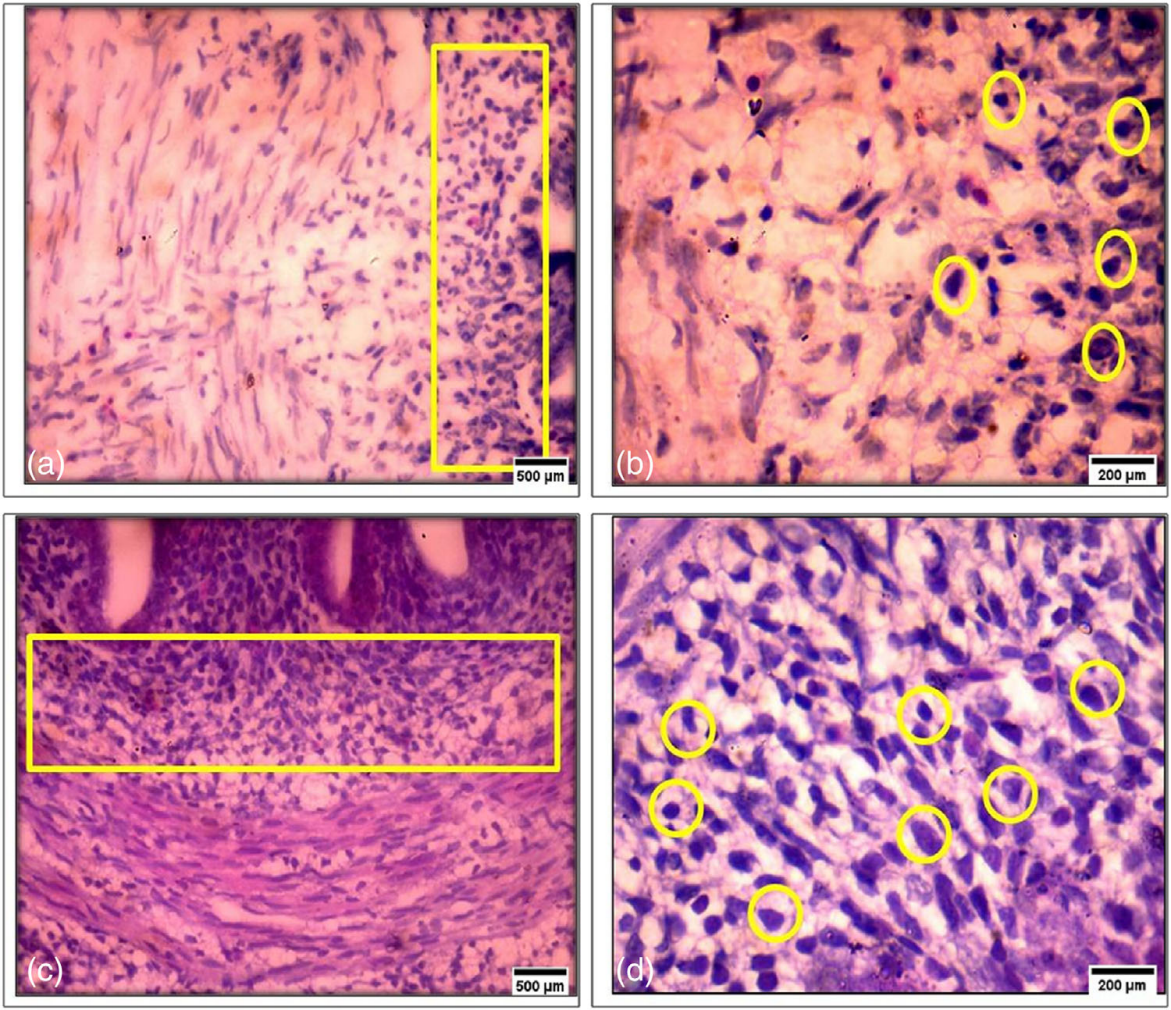
14‐day chick embryo caecal tonsil histology is shown. (a) The 4G radiation‐exposed group had low‐density lymphocytes in lamina propria (in rectangle) (haematoxylin and eosin [H&E], 40×, 500 μm). (b) The 4G radiation‐exposed group has decreased lymphocytes in lamina propria (circles) (H&E, 100×, 200 μm). (c) The control group without exposure has high‐density lymphocytes in lamina propria (in rectangle) (H&E, 40×, 500 μm). (d) The control group without exposure has increased lamina propria lymphocytes (circles) (H&E, 100×, 200 μm).

At day 14 of the chicken embryo, the exposed group (Figure 7a,b) exhibited a decrease in the number of lymphocytes in the caecal tonsil compared to the control group (Figure 7c,d). The numerical representation of the caecal tonsils from both the control group and the group exposed to 4G is presented in Table 3. The total lymphocyte count in the control group was significantly higher (147.2 ± 9.06) than in the exposure group (86.8 ± 5.38) (*p* < 0.05).

**TABLE 3:** Comparison of lymphocyte count in caecal tonsil between exposure group (A) and control group (B).

Group	Exposure (A)	Control (B)
Number of lymphocytes	86.8 ± 5.38^*^	147.2 ± 9.06^*^

*Note*: *n* = 5 for each group.

* *p* < 0.05 statistically significantly.

### 3.7 Vascular gene expression

**FIGURE 8:**
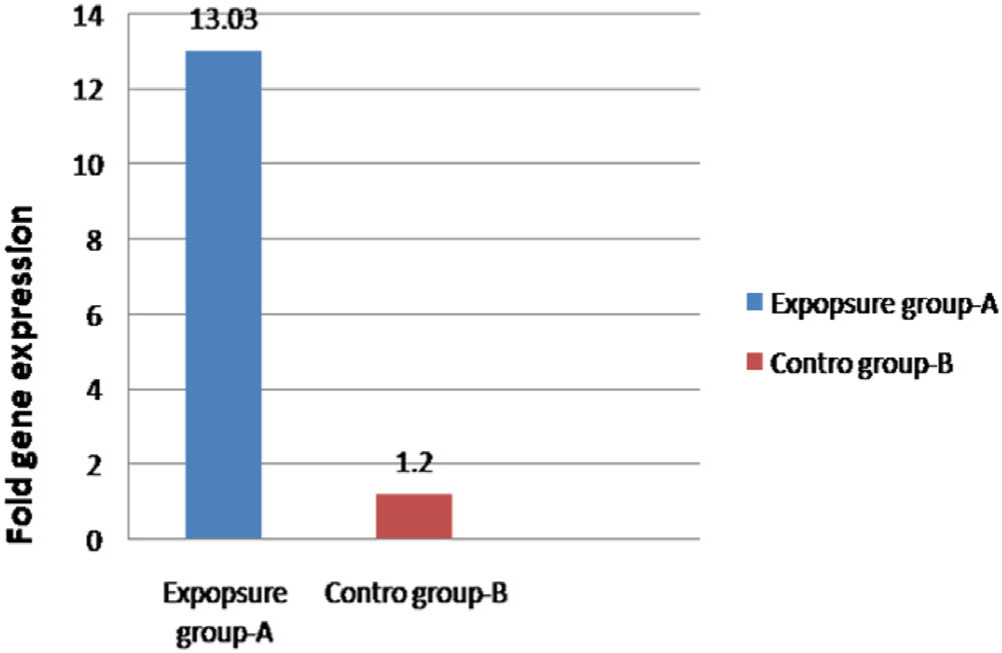
The figure shows the effect of 4G (2100 MHz) cell phone radiation on the expression of the vascular gene *VEGF‐A* in chicken embryos. The expression of the *VEGF‐A* gene (*n* = 3 per group) was observed to increase significantly in the exposure group compared to the control group.

On the 14th day of the incubation period, the expression of the vascular gene VEGF‐A was evaluated using the qPCR test. As illustrated in Figure 8, exposure to 4G cell phone radiation resulted in a significant increase in the mRNA expression of the VEGF‐A gene when compared to the control.

### 3.8 Immunity gene expression

**FIGURE 9:**
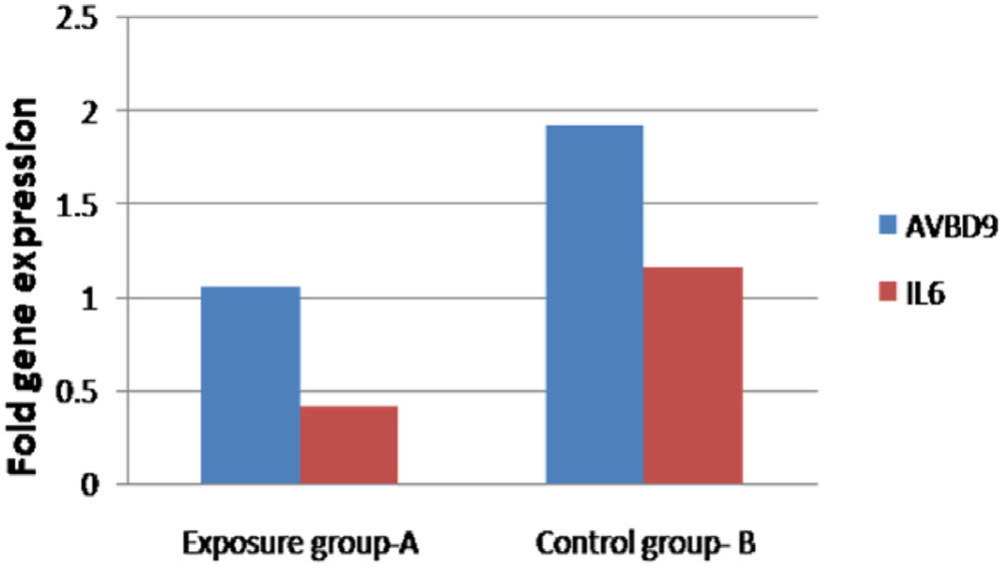
The figure shows the impact of 4G (2100 MHz) cell phone radiation on the expression of immunogenic genes *AvBD9* and *IL6* in chicken embryos. The expression of *AvBD9* and *IL6* genes (*n* = 3 per each group) decreased significantly in response to 4G cell phone radiation exposure compared to the control group.

On day 14 of the incubation period, the expression of immunity genes AvBD9 and IL6 was evaluated using qPCR. The results revealed a significant decrease in the mRNA expression of AvBD9 and IL6 in the exposure group to 4G cell phone radiation compared to the control group (Figure 9).

## 4 DISCUSSION

Despite the increasing number of reports on the effects of cell phone radiation on human health, there is currently no conclusive evidence to support these claims. However, it is believed that the biological changes observed may result from exposure to electromagnetic radiation. Various researchers have reported different findings due to several factors, such as frequency, intensity, field variations and the degree of exposure (Gye & Park, 2012). Cell phones that use the Global System for Mobile Communications operate within the frequency band of 300 MHz to 300 GHz (Dubreuil et al., 2002). In Bangladesh, the International Telecommunication Union Radio Regulation and National Fire Protection Association have reserved frequency bands of 2100 to 2690 MHz for 4G services. This study exposed the egg embryos to 2100 MHz (4G Grameen phone network) frequency. Cell phones are allowed a maximum SAR of 2 W/kg; however, most smartphones have a SAR rating of only 1.4 W/kg (Agarwal et al., 2011). For this study, a SAR value of 1.4 W/kg was assigned to the exposure group.

This study's experimental incubation period, from days 1 to 14, did not cause any deaths or adverse effects in the control or experimental groups. The exposed group's weight and length were much lower than in the control group. Radiofrequency radiation (RFR) creates free radicals at the cellular level and damages DNA at the molecular level, causing 4G group embryos to grow less. RFR can accelerate or decrease cell proliferation, especially during embryonic development (Panagopoulos, 2011; Zareen et al., 2009). Mobile radiation at certain frequencies can inhibit growth (Amer et al., 2013; Marcickiewicz, 1986; O'Connor, 1999). Intrauterine RFR radiation from 27.12 to 2450 MHz lowered mice’ foetal weight and crown‐rump length (Bonhomme‐Faivre et al., 2003). This investigation found just subcutaneous haemorrhage. The study did not find monophthalmia, spina bifida, microphthalmia, exencephalic embryos, anophthalmia, shorter beaks or asymmetrical faces, as described by Lahijani et al. (2009).

The exposed group had significantly higher urea and CT levels than the control group. Asgari et al. (2014) and Eslami et al. (2019) found that cell phone electromagnetic radiation raises urea and CT levels. Cell phone radiation also causes metabolic alterations and liver and kidney oxidative stress (Eslami et al., 2019). The amniotic fluid of radiation‐exposed chicken embryos had higher ALP, AST and ALT values than the control group. Ghaedi et al. (2013) and Sani (2018) found similar results. Mobile phone radiation harms the liver, kidneys, heart and brain (Hasan et al., 2022). We found cytoplasmic vacuolations in embryonic hepatocytes exposed to 4G radiation, similar to prior research. Lahijani et al. (2009) found damaged hepatocytes, fibrotic liver bands and abnormal lipid deposition in white leghorn chicken fetuses subjected to 50 Hz electric radiation, which pushed hepatocyte nuclei to the cell's periphery. Our investigation found no necrotic changes. Khalil et al. (2012) saw no histological changes in Balb/c mice exposed to 900 MHz mobile radiation for 30 min a day for a month. This study suggests cell phone radiation can alter biochemical and histological markers and impair foetal development.

Radio waves at 900–2100 MHz have been shown to affect the nervous system in several investigations. Radio waves of 900 MHz caused pyknotic neurons in the hippocampus, deeply stained granule cells in the cerebrum, dark cytoplasm and pyramidal neurons (Bas et al., 2013; Odaci, 2008; Sahin et al., 2015). Degenerated neurons displayed shrinking cell bodies and darkly pigmented nuclei in our investigation. Hasan et al. (2022) found that exposed embryos had fewer neuron cells. Radiation alters antioxidant defence mechanisms and inflammation, which increases ROS production and oxidative stress. Radiation exposure causes tissue oxidation in several studies. Radiation increases superoxide dismutase, catalase and malondialdehyde levels. Apoptosis and fewer neurons may result from this enzyme increase (Dasdag, 2008; Kesari, 2010; Misa‐Agustino, 2015; Ozguner et al., 2005).

The results of this study revealed that the exposed group had markedly higher levels of VEGF‐A expression than the control group. Consistent with the Regenfuss and Cursiefen (2010) findings, VEGF‐A can be secreted in response to hypoxia, inflammation and low blood sugar. The observed increase in VEGF‐A expression in the inflamed regions could be attributed to the oxidative stress and inflammation induced by the 4G radiation exposure.

Islam et al. (2012) found no significant development occurred until day 14 of the chicken embryo when lamina propria lymphocytes were observed in the caecal tonsils. The study focused on analysing chicken embryo's caecal tonsils on day 14. The results revealed that the number of lymphocytes was comparatively reduced in the exposed group compared to the control group. Furthermore, the expression of two immunity genes, IL6 and AvBD9, was significantly reduced in the exposed group compared to the control group. One possible explanation for these findings is that EMF from cell devices may interfere with the regulation of Ca^2+^ in lymphocytes (Walleczek et al., 1992) or prolong the free radicals’ half‐life and increase the reactive levels of oxygen species within cells (Balci et al., 2007).

According to Lee et al. (2004), oxidative stress from exposure to RF‐EMFs can cause damage to essential cell components, such as lipids and nucleic acids. Moreover, Lantow et al. (2006) demonstrated that mammalian macrophages and lymphocytes produced significantly more reactive oxygen species upon exposure to 1800 MHz RF‐EMFs, leading to increased free radicals and disrupted Ca^2+^ regulatory mechanisms that can impair cell development, resulting in protein misfolding and damaged DNA (Gye & Park, 2012).

In a study by Paulraj et al. (2006), rats exposed to reduced microwaves at frequencies of 2.45 and 16.5 GHz with SARs of 1.0 and 2.01 W/kg, respectively, for 25 days showed a statistically significant (*p* < 0.001) rise in DNA single‐strand breaks in brain cells. Cells cannot fully repair such breaks in the DNA double‐ or single‐strand structure and DNA chemical bonds (Robison et al., 2002). Although the mass of DNA lesions is restored after a few hours or days, long‐term occupational exposure increases DNA damage and reduces the healing ability. Garaj‐Vrhovac et al. (2009) demonstrated that some DNA damage caused over time persists, even after the mass of DNA lesions is restored. Thus, exposure to radio waves can cause DNA damage to accumulate over time, leading to potentially harmful effects on cellular functions and health.

## 5 CONCLUSION

In conclusion, 4G mobile phone radiation causes growth redundancy, pathological abnormalities in key organs and immune and vascular gene expression modifications. These results question mobile phone radiation safety standards and imply that 4G radiation sensitivity may vary. The study highlights the need for more detailed investigations into specific gene expression and organ pathology changes to unravel the underlying mechanisms of cellular and tissue damage. The health consequences of chronic 4G mobile phone radiation exposure must be investigated. Robust epidemiological studies and controlled experiments on human subjects are necessary to comprehensively understand the risks involved. Mobile phone radiation's long‐term effects, primarily on fetuses and children, must be studied and safety precautions taken. The parallel to cigarettes emphasizes the need for comprehensive research to assess the chronic consequences of mobile phone radiation, including carcinogenicity, and to establish safety recommendations. Given the growing popularity and possible exposure to 5G technology, future studies should also include it. According to these results, cell phones are unsafe, especially for children and pregnant women. We can safeguard people and build safer technology by filling these research gaps and learning about mobile phone radiation.

## AUTHOR CONTRIBUTIONS

*Conceptualization; data curation; methodology; investigation; supervision; writing – original draft; writing – review and editing*: Md. Sadequl Islam. *Conceptualization; investigation; writing – original draft; writing – review and editing*: Md. Mominul Islam. *Conceptualization; methodology; writing – review and editing*: Md. Moshiur Rahman. *Data curation; investigation; methodology; writing – review and editing*: Khaleda Islam.

## CONFLICT OF INTEREST STATEMENT

The authors affirm that there are no conflicts of interest related to this work.

## FUNDING INFORMATION

Institute of Research and Training (IRT), Hajee Mohammad Danesh Science and Technology University, Dinajpur‐5200, Bangladesh, (Grant Number: HSTU/IRT/3358/2021/110) and Ministry of Science and Technology, Government of the Peoples Republic of Bangladesh (grant Number: R&D/39.00.0000.012.002.07.21.62.56.86)

## ETHICS STATEMENT

The Ethical Committee of the Institute of Research and Training (IRT) at Hajee Mohammad Danesh Science and Technology University has approved the strategy (Ref. No. HSTU/IRT/3909) for maintaining embryos in laboratory research and collecting samples.

### PEER REVIEW

The peer review history for this article is available at https://publons.com/publon/10.1002/vms3.1273.

## Data Availability

On request, the corresponding author will provide access to the data used in this research.
